# Using Omniscan-Loaded Nanoparticles as a Tumor-Targeted MRI Contrast Agent in Oral Squamous Cell Carcinoma by Gelatinase-Stimuli Strategy

**DOI:** 10.1186/s11671-019-3214-5

**Published:** 2019-12-30

**Authors:** Antian Gao, Yuehui Teng, Pakezhati Seyiti, Yingtzu Yen, Hanqing Qian, Chen Xie, Rutian Li, Zitong Lin

**Affiliations:** 1 Department of Dentomaxillofacial Radiology, Nanjing Stomatological Hospital, Medical School of Nanjing University, 30 Zhongyang Road, Nanjing, 210008 China; 2 Central Laboratory of Stomatology, Nanjing Stomatological Hospital, Medical School of Nanjing University, No 22 Hankou Road, Nanjing, 210093 China; 3 The Comprehensive Cancer Center of Drum-Tower Hospital, Medical School of Nanjing University & Clinical Cancer Institute of Nanjing University, 321 Zhongshan Road, Nanjing, 210093 China; 4 Key Laboratory for Organic Electronics and Information Displays & Jiangsu Key Laboratory for Biosensors, Institute of Advanced Materials (IAM), Jiangsu National Synergetic Innovation Center for Advanced Materials (SICAM), Nanjing University of Posts & Telecommunications, Nanjing, 210023 China

**Keywords:** Tumor-targeted MRI contrast agent, Nanoparticles, Omniscan, Gelatinase-stimuli, Oral squamous cell carcinoma

## Abstract

In this study, the tumor-targeted MRI contrast agent was prepared with gelatinase-stimuli nanoparticles (NPs) and Omniscan (Omn) by double emulsion method. The size, distribution, morphology, stability, drug loading, and encapsulation efficiency of Omn-NPs were characterized. The macroscopic and microscopic morphological changes of NPs in response to gelatinases (collagenases IV) were observed. The MR imaging using Omn-NPs as a contrast agent was evaluated in the oral squamous cell carcinoma models with Omn as a control. We found clear evidence that the Omn-NPs were transformed by gelatinases and the signal of T1-weighted MRI sequence showed that the tumor-to-background ratio was significantly higher in Omn-NPs than in Omn. The peak point of time after injection was much later for Omn-NPs than Omn. This study demonstrates that Omn-NPs hold great promise as MRI contrast agent with improved specificity and prolonged circulation time based on a relatively simple and universal strategy.

## Introduction

Oral squamous cell carcinoma (OSCC) is the most common malignant tumor in oral and maxillofacial region; due to the special location of OSCC, the surgical treatment inevitably affects the functions and esthetics of the orofacial region. Early and accurate diagnosis of OSCC allows more individual and proper surgical treatment and therefore, resulting in less morbidity following treatment as well as better patient prognosis. Correct diagnosis and staging, which affects treatment planning for the disease, requires the use of imaging techniques [1].

MRI is a non-invasive imaging modality with no ionizing radiation. It may be utilized to provide high-resolution and three-dimensional images of soft tissues. Multiparametric MRI has been tested in clinical trials and is proven to be useful in tumor localization [2]. While various compounds have been evaluated as MRI contrast agents, gadolinium (Gd) complexes continue to be the most widely used and accounting for essentially all of the agents applied in the clinic at present [3]. However, the existing Gd-based MRI contrast agents are not tumor-specific and unable to provide precise detection and characterization of tumor. Due to the small size, most of these agents are able to be distributed in intravascular and interstitial spaces and rapidly discharged through renal filtration [3]. To improve the specificity in tumor tissues and prolong circulation time in the blood flow of the MRI contrast agent, researchers have tried to design and synthetize variable new MRI contrast agents [4–8].

Recent past, many methoxy-poly(ethylene glycol)(mPEG) and/or polycaprolactone (PCL)-related nanoparticles (NPs) were designed and studied [9–11]. These NPs were used to delivery drugs, they aided drug solubility, improved the therapeutic process by extending the circulation time and enhancing uptake into tumors, through the enhanced permeability and retention effect. mPEG and PCL are US Food and Drug Administration approved copolymer which exhibits very low immunogenicity, antigenicity, and toxicity, and are widely studied for medical applications [12]. It is known that the biocompatibility and biodegradability are important properties when NPs are used in the field of medical, environmental, and chemical engineering [13, 14]. In our previous work, we have developed a gelatinase-stimuli drug delivery system based on the mPEG and PCL with tumor-specific gelatinases-cleavable peptide inserted between mPEG and PCL [12]. Therapeutic drugs such as docetaxel, miR-200c were loaded on this nanoparticle. The in vitro and in vivo studies showed that the drugs could be delivered specifically into the tumor tissues [15]. Our NPs are based on a relatively simple tumor-targeted strategy. The enhanced permeability and retention (EPR) effect could accumulate the nanoparticles into tumor tissues. Gelatinases (matrix metalloproteases-2/9 MMP2/9, collagenases IV), which are widely expressed in tumors, would separate the NPs and release the loaded drugs. Unlike the active targeting strategy, our NPs have the potential of loading variable therapeutic and diagnostic drugs, which would be more simple and universal.

In this study, we loaded the same type of NPs with Omn, a widely used MRI contrast agent [16], to achieve the goal of establishing a tumor-targeted, biocompatible, and biodegradable MRI contrast agent. The effectiveness of Omn-NPs as MRI contrast agent was evaluated in the xenograft model of human oral squamous cell carcinoma with Omn alone as a control.

## Materials and Methods

### Materials

Methoxy-polyethyleneglycol-NHS (mPEG-NHS, Mn 5000) was purchased from Beijing Jiankai Technology Co (Beijing, China). The gelatinase-cleavable peptide (sequence: H2N-PVGLIG-COOH) was synthesized by Shanghai HD Biosciences Co (Shanghai, China). Omniscan (Gadodiamide Injection) was purchased from GE Healthcare (Ireland). Collagenases IV were purchased from Sigma (USA).

### Synthesis of Omn-Loaded Gelatinases-Stimuli NPs

Gelatinases-cleavable copolymer mPEG-Pep-PCL and mPEG-PCL without peptide were synthesized by ring opening copolymerization as the same in our previous work [17]. The Omn-NPs were formulated by double emulsion solvent evaporation technique. Briefly, 10 mg of mPEG-Pep-PCL copolymer was dissolved in 1 mL dichloromethane (DCM). Then, 0.1 mL, 0.2 mL, and 0.3 mL Omn were added respectively. This mixture was emulsified in 3 mL of 3% (w/v) aqueous polyvinyl alcohol (PVA) solution by sonication (XL2000, Misonix, Farmingdale, NY, USA) for 60 s to obtain an oil/water (o/w) emulsion. This emulsion was then emulsified in 5 mL aqueous solution containing 0.5% (w/v) by sonication PVA for 60 s. The w/o/w emulsion formed was gently stirred at room temperature in a fume hood until the organic solvent had evaporated. The resulting solution was filtered to remove non-incorporated drugs. Blank-NPs were prepared with the same manner described, without adding Omn. Omn-loaded mPEG-PCL NPs (Con-Omn-NPs) were synthesized with 10 mg of mPEG-PCL copolymer and 0.2 mL Omn followed the same steps.

### Particle Size Measurement and Morphology Examination of NPs

The particle sizes and stability of Omn-NPs and blank-NPs were measured by dynamic light scattering (DLS) (Brookhaven Instruments Corporation, USA). Omn-NPs and blank-NPs were kept at room temperature. Particle sizes were determined by DLS every 2 days to evaluate the stability of the Omn-NPs (totally for 6 days). The values were the average of triplicate measurements for a single sample. Morphology examination of Omn-NPs and blank-NPs was conducted using a transmission electron microscope (TEM) (JEM-100S, JEOL, Japan). One drop of properly diluted NPs suspension was placed on a copper grid covered with nitrocellulose membrane and air-dried at room temperature. The sample was negative stained with phosphotungstic sodium solution 1% (w/v) before observation.

### The Drug Loading Content and Encapsulation Efficiency

The drug loading content and encapsulation efficiency of Omn-NPs were analyzed by calculating the concentration of gadolinium ion. One milliliter of Omn-NPs was split by concentrated nitric acid, and then the mixture was diluted by dilute nitric acid. The sample was tested by Inductively Coupled Plasma-Atomic Emission Spectrometry (ICP-AES, Optima 5300DV, PerkinElmer, USA).

$$ \mathrm{Drug}\ \mathrm{loading}\ \mathrm{content}\%=\frac{\mathrm{Wight}\ \mathrm{of}\ \mathrm{the}\ \mathrm{drug}\ \mathrm{in}\ \mathrm{nanoparticals}}{\mathrm{Weight}\ \mathrm{of}\ \mathrm{the}\ \mathrm{nanoparticals}} $$

$$ \mathrm{Encapsulation}\ \mathrm{efficiency}\%=\frac{\mathrm{Weight}\ \mathrm{of}\ \mathrm{the}\ \mathrm{drug}\ \mathrm{in}\ \mathrm{nanoparticals}}{\mathrm{Weight}\ \mathrm{of}\ \mathrm{the}\ \mathrm{feeding}\ \mathrm{drug}} $$

### Macroscopic Changes and Microscopic Morphological Changes of NPs in Response to Collagenase

Omn-loaded mPEG-PCL NPs (Con-Omn-NPs) and mPEG-Pep-PCL NPs (Omn-NPs) were incubated with Hank’s solution containing collagenase IV (0.34 mg/mL) at 37 °C for 24 h. Changes in solution transparency were observed by the naked eyes.

Microscopic morphology evaluation of Con-Omn-NPs and Omn-NPs (incubated with or without collagenase) was conducted using a TEM. For TEM, one drop of NPs suspension was placed on a copper grid covered with a nitrocellulose membrane and air-dried prior to observation.

### In **V**itro **C**ellular **U**ptake

Human oral squamous cell carcinoma lines (HSC3) were kindly provided by the Ninth Hospital of Shanghai. The tumor cells were seeded in a 24-well plate at a density of 5 × 10^5^ cells per well and cultured for 24 h. Then Coumarin-6-loaded mPEG-Pep-PCL NPs (12.5 μg/mL calculated by coumarin-6) were added into the cultured medium and incubated for 0.5 and 1 h at 37 °C. Cultured medium was sucked out and washed three times with PBS. Cells were immobilized for 20 min with absolute ethanol (1 mL per well), then washed three times by PBS. The cells were observed by immunofluorescent cytochemistry and confocal laser scanning microscope (LSM710, Carl Zeiss MicroImaging GmbH, Berlin, Germany). The excitation and emission wavelength was 460 nm for coumarin-6.

### Animals

All animal experiments were performed in full compliance with guidelines in the Guide for the Care and Use of Laboratory Animals published by the US national Institutes of Health (NIH publication No.85-23, revised 1985) and was approved by the Ethics Review Board for Animal Studies of Nanjing Stomatological Hospital, Medical School of Nanjing University. BALB/c mice (5–6 weeks, 18–22 g) were purchased from Model Animal Research Center of Nanjing University. Animal health, including body weight and skin conditions, was monitored twice weekly. Ulceration, a reduction in animal mobility and weight loss, was not observed during the experiment.

### OSCC Model Establishment

The tumor cells were cultured in Dulbecco’s modified Eagle’s medium (DMEM) with 10% fetal bovine serum (FBS), 100 U/mL penicillin, and 100 mg/mL streptomycin at 37 °C in a humidified atmosphere containing 5% CO_2_ and 95% air. To establish a xenograft model of human OSCC, the human OSCC cells HSC3 (1 × 10^6^ cells in 50 μL phosphate buffer saline (PBS)) were subcutaneous inoculated into the right armpit of nude mice (3 mice per group). We measured the tumor dimension every other day by a caliper. When the tumor diameter was approximately 0.4–0.5 cm, the mice were ready for in vivo MR imaging experiments.

### In Vivo MRI Study with Omn-NPs and Omn as Contrast Agent

For in vivo study, we divided the mice into two groups (A and B). Mice in group A were injected Omn-NPs through tail vein while mice in group B were injected with the same concentration of Omn as the NPs loaded. Both of the groups were scanned using Bruker Biospin 7.0 T MRI scanner (Bruker BioSpin, Ettlingen, Germany). The parameters were set as follows: field of view (FOV), 3.5 × 2.5 cm; slice thickness, 0.8 mm; TR, 745.2 ms; TE, 7.5 ms. The axial slices of mouse were acquired using T1-weighted spin echo sequence. Images were obtained before and at different time points after intravenous administration of two contrast agents.

### Expression of MMP2/9 in Tumor and Normal Tissues

After in vivo MRI examination, the tumor tissues, heart, liver, spleen, lung, kidney, and muscle tissues from OSCC mice model were selected for immunohistochemical (IHC) staining for MMP2 and MMP9. All the tissues were dissected and fixed in 10% neutral buffered formalin, routinely processed into paraffin, and sectioned at a thickness of 5 μm. IHC examination for the semi-quantitative expression (−, +, and ++) of MMP2/9 was conducted using optical microscopy.

### Statistical Analysis

Statistical analysis was performed using Student’s *t* test. The data were listed as mean ± SD, and a value of *p* < 0.05 was considered statistically significant.

## Results and Discussion

### Characterization of mPEG-Pep-PCL Nanoparticles

**Fig. 1:**
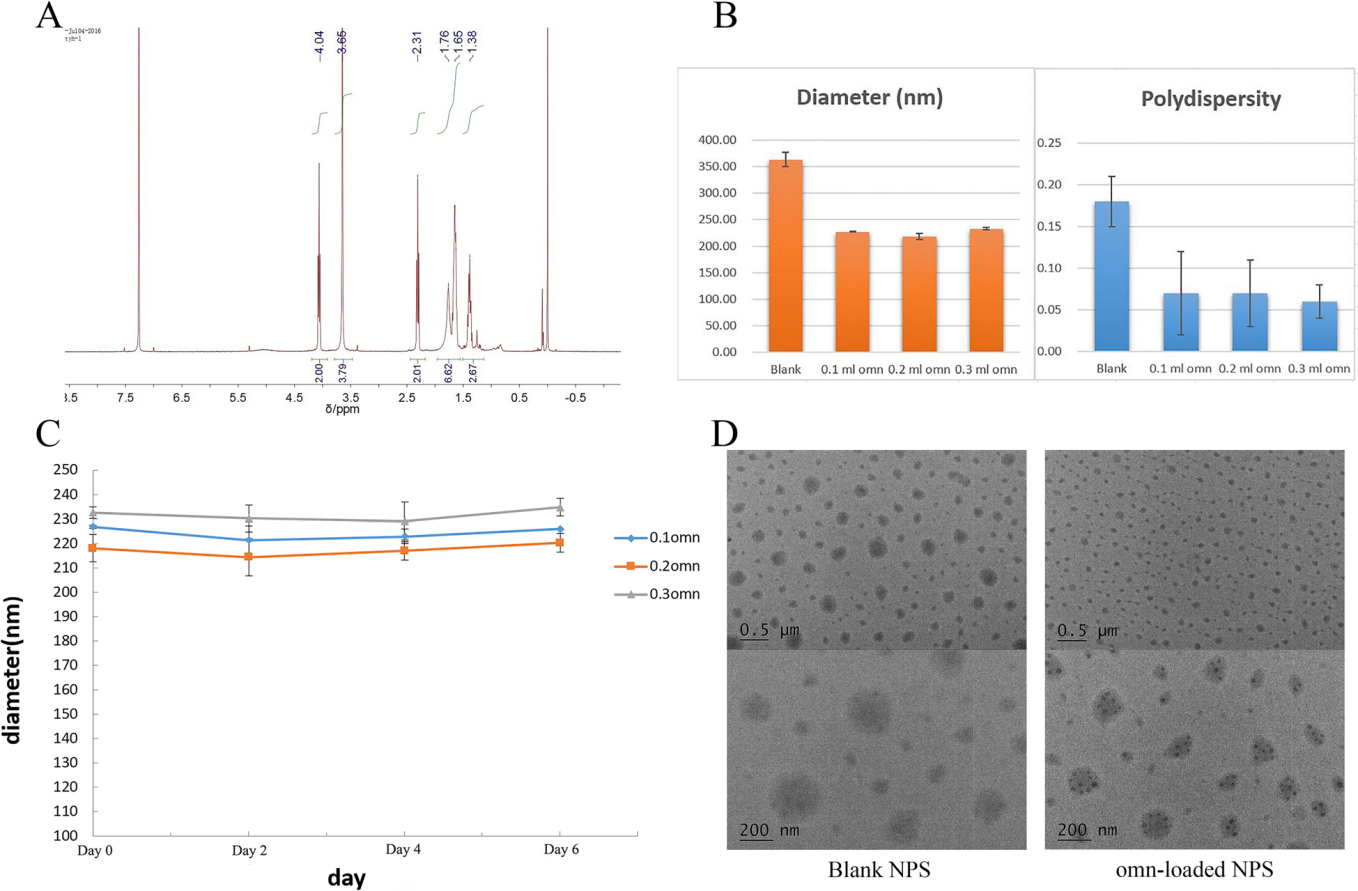
**a** ^1^H nuclear magnetic resonance spectra (300 MHz, 25 μC) of PEG-Pep-PCL in CDCl3. **b** The diameter and polydispersity index of blank-NPs and Omn-loaded NPs (0.1 mL, 0.2 mL, 0.3 mL). **c** The stability of Omn-loaded NPs (0.1 mL, 0.2 mL, 0.3 mL). **d** TEM micrographs of blank-NPs and Omn-loaded NPs. The error bars represent the standard deviations of three separate measurements

The ^1^H NMR(CDCl_3_)spectra of mPEG-Pep-PCL copolymers confirmed that the peptide was successfully conjugated with mPEG and the mPEG-Pep conjugates were successfully conjugated with PCL (Fig. 1a). The mole ratio of hydrophilic block to hydrophobic block (mPEG/PCL) in mPEG-Pep-PCL copolymer was about 0.95 based on the integral ratio of -CH2-O- (4.04 ppm) in PCL segment to -CH2-CH2-O (3.65 ppm) in mPEG segment from ^1^H NMR measurement.

### Particle Sizes and Stability of NPs

The particle sizes and polydispersity index (PDI) were determined by DLS (Fig. 1b). No significant differences in particle sizes among the three Omn-NPs were found (*p* > 0.05), while significant differences were found between Omn-NPs and blank-NPs (*p* < 0.05). For the PDI, no significant differences were found among these Omn-NPs (*p* > 0.05), but there were significant differences between Omn-NPs and blank-NPs (*p* < 0.05).

For the stability of Omn-NPs, no precipitation and obvious change in sizes were observed in all the three Omn-NPs (Fig. 1c), which indicated that Omn-NPs were stable.

### Morphological Studies of NPs

The TEM micrographs of blank-NPs and Omn-NPs are presented in Fig. 1d. The oblate shape could also be observed in both blank-NPs and Omn-NPs and the Omn-NPs were much smaller than blank-NPs due to their different sizes. Moreover, the Omn in the NPs could be clearly distinguished, which appeared as dark particulate in the NPs. This Omn particulate could be observed dispersedly within the NPs.

### Drug Loading Content and Encapsulation Efficiency

The drug loading content and encapsulation efficiency of the three Omn-NPs are shown in Table 1. The result showed that 0.3 mL Omn had the highest drug loading but encapsulation efficiency was quite low, and 0.1 mL Omn had the highest encapsulation efficiency and relatively close drug loading with 0.2 mL and 0.3 mL Omn. Considering both the drug loading and encapsulation efficiency, 0.1 mL Omn-NPs were used in the final in vivo MRI study. The low encapsulation efficiency also indicated that in the reaction system, the Omn we added was sufficient for NPs.

**Table 1:** Drug loading content and drug encapsulation efficiency of the three Omn-NPs

Nanoparticles	Drug loading content (%)	Drug encapsulation efficiency (%)
0.1 mL Omn-NPs	10.35	4.03
0.2 mL Omn-NPs	11.87	2.35
0.3 mL Omn-NPs	15.64	2.16

### Macroscopic and Microscopic Morphological Changes of Omn-NPs and Con-Omn-NPs in Response to Collagenase IV

**Fig. 2:**
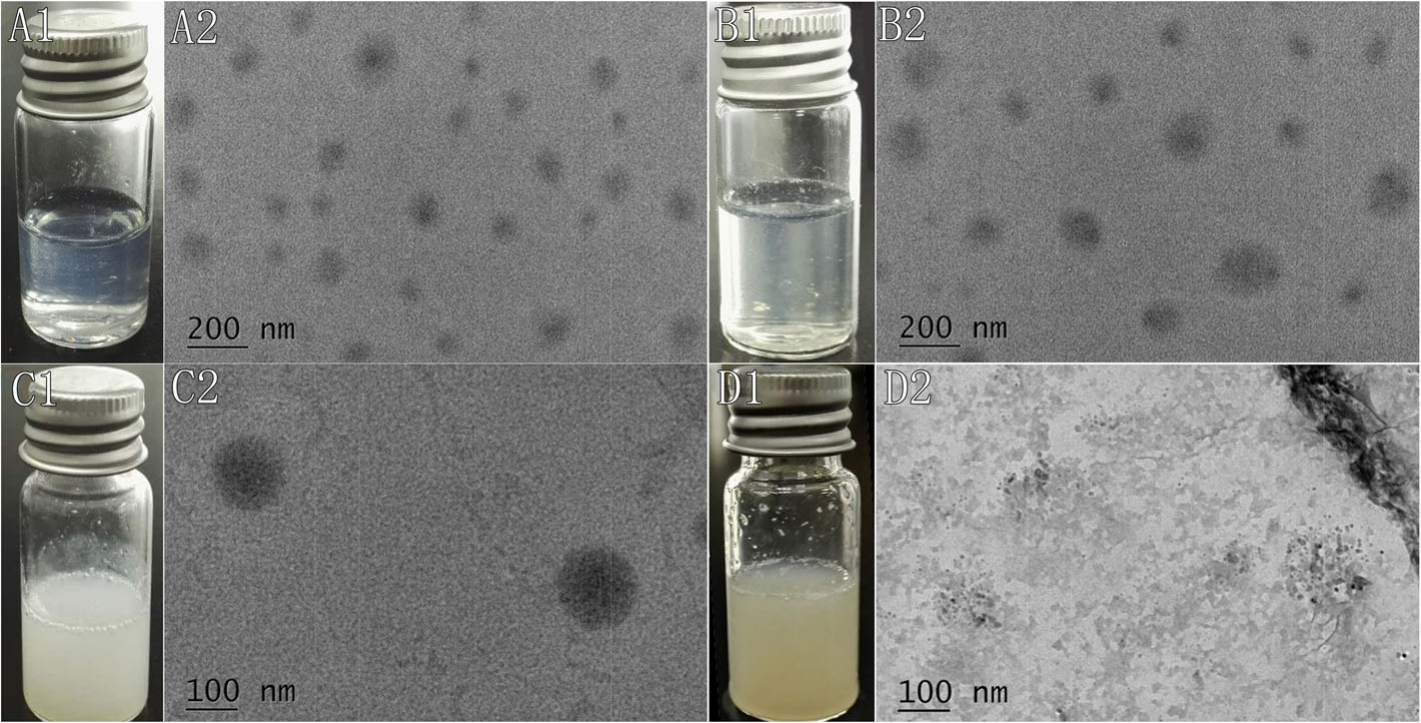
**a1**, **a2**, **b1**, **b2**, **c1**, **c2**, **d1**, **d2** Macroscopic and microscopic morphological change of Omn-loaded mPEG-PCL NPs (Con-Omn-NPs) and Omn-loaded mPEG-Pep-PCL NPs (Omn-NPs) after incubation with collagenase IV

To verify cleavage of NPs in response to gelatinase (collagenase IV), the macroscopic and microscopic morphological changes of Omn-NPs and Con-Omn-NPs after incubation with Hank’s solution containing 2 mg/mL collagenase IV were evaluated. A1 and B1 showed the transparent solutions of Con-Omn-NPs before and after incubation with collagenase IV, and A2 and B2 showed no change was found for the microscopic morphology of Con-Omn-NPs using TEM before and after incubation. C1 and D1 showed the solutions of Omn-NPs before and after incubation with collagenase IV. D1 showed that the liquid turned turbid as precipitation occurred in the Omn-NP solutions after 24 h. D2 showed the TEM images of Omn-NPs in response to collagenases IV, the structures of NPs were break down (Fig. 2). This result indicated our NPs were gelatinase-stimuli: the cleavage of the peptide would break up the NPs, and the loaded drugs would be released. And the feature of cleaving the peptide to release the loaded drugs was also demonstrated via drug release and in our previous research [12, 18].

### In Vitro Cellular Uptake Studies

**Fig. 3:**
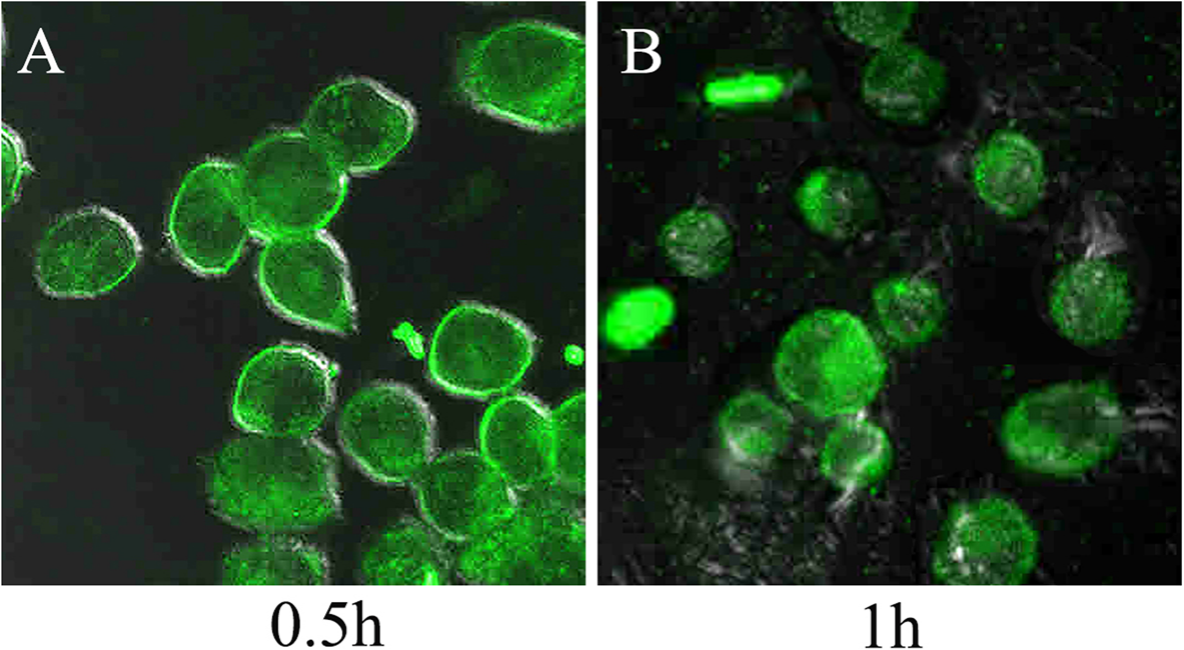
**a, b** In vitro HSC3 cellular uptake studies of nanoparticles. Confocal microscopy images of HSC3 cells after incubation with coumarin-6-loaded NPs

The cellular uptake of coumarin-6-loaded NPs is shown in Fig. 3. The green fluorescence from coumarin-6 was showed in cytoplasm of the HSC3 cells, suggesting that coumarin-6 entered cytosol together with NPs. As coumarin-6 was originally entrapped in the NPs, which indicated our NPs could effectively penetrate cell membrane barriers and distribute in cell cytoplasm via endocytosis.

### MR Imaging In Vivo with Omn-NPs and Omn as Contrast Agents

**Fig. 4:**
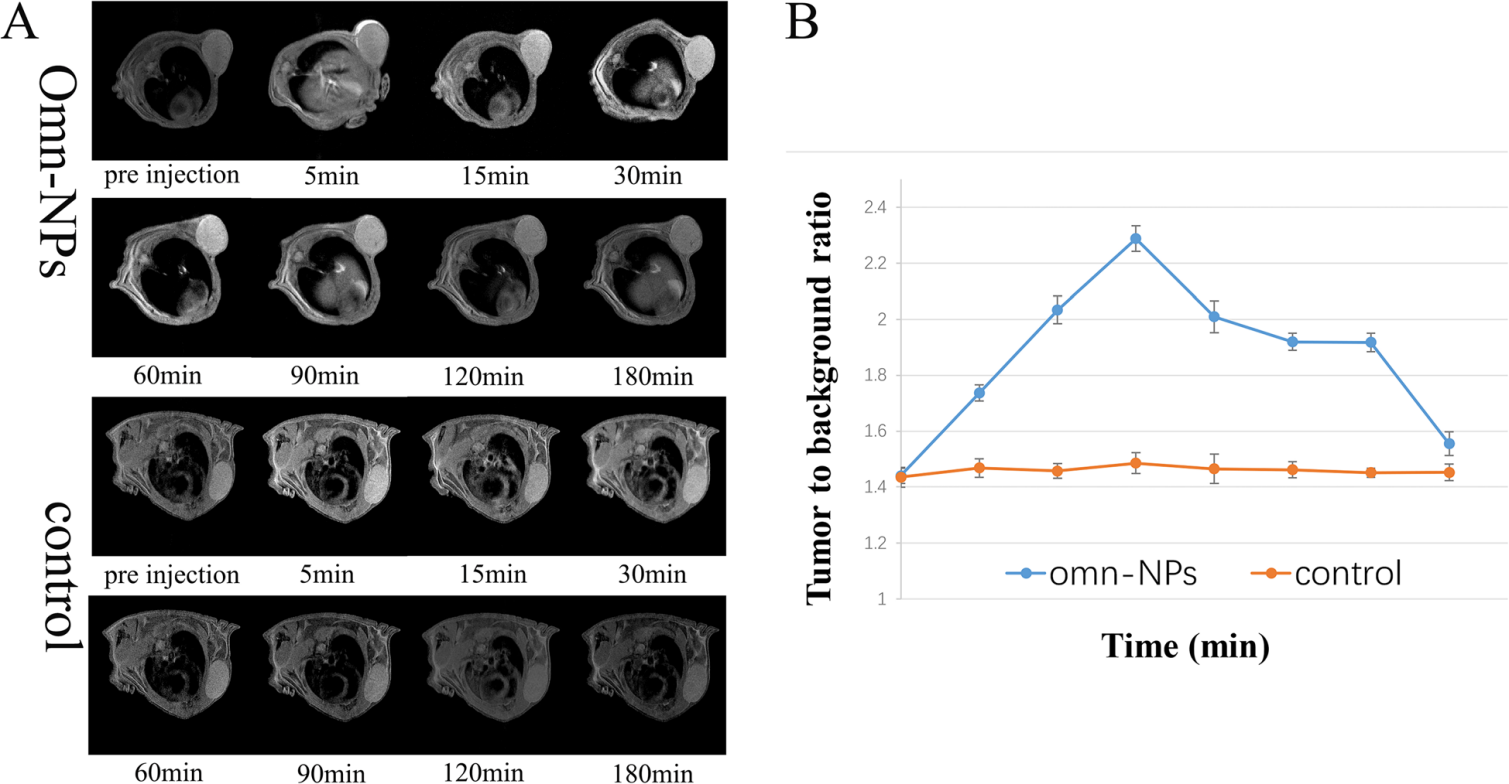
**a**, **b** Axial MRI images and line chart of tumor-to-background ratio of xenograft model of human OSCC at the indicated tumor positions acquired with a T1-weighted sequence. The error bars represent the standard deviations of three separate measurements

Images were acquired before Omn-NPs and Omn administered intravenously at the dose of 0.025 mmol/kg (Gd^3+^) of 2 groups. Post-contrast images were then obtained at 5 min, 15 min, 30 min, 60 min, 90 min, 120 min, 150 min, and 180 min after injection (Fig. 4a).The signal of tumor-to-background (TBR) ratio was calculated and used as a quantifiable indicator for evaluation using Omn-Nps compared with Omn. The results showed that maximum TBR for Omn-NPs was 2.23 ± 0.10 and 1.48 ± 0.01 for Omn, the time to peak was 30 min for Omn-NPs and 5 min for Omn, and the signal enhancement duration time was 180 min for Omn-NPs and 30 min for Omn (Fig. 4b). There was a significant difference for maximum TBR and retention time between the two groups (*p* < 0.05). Although our Omn-NPs had relatively low drug loading, superior enhanced in vivo MR imaging was demonstrated compared with Omn alone. This also proved that our Omn-NPs were gelatinase-stimuli and tumor-specific.

Despite recently advanced imaging techniques such as single-photon emission computed tomography [19], positron emission tomography (PET) [20], and optical image [21] were used in the diagnosis of OSCC, MRI is still the most widely used and reliable tools for staging head and neck tumors according to the TNM cancer staging system [1] and gadolinium chelates are still the most widely used MRI contrast agents [22].

To achieve the goal of targeting tumor specifically for MRI contrast agent, active and passive targeting strategies have been used [2, 23, 24]. Active targeting [12] has become a large area of focus in cancer diagnosis. Targeting ligands, such as aptamer [25], peptide [8], antibody [6], and folate [26], are conjugated to macromolecular and supramolecular multimeric Gd complexes for binding to particular receptors overexpressed by tumor cells or vasculatures. However, the biocompatibility and biodegradability properties of these macromolecular and supramolecular are not clear, and their non-biodegradability hinders the clinic application. However, our Omn-NPs consist of PEG, PCL, gelatinase-cleavage peptide, and Omn. The PEG and PCL have excellent biocompatibility and biodegradability properties, and Omn is a clinically widely used MRI contrast agent; therefore, our Omn-NPs are expected to have excellent biosafety.

The modification of polymer with hydrophilic mPEG could prolong the blood circulation and increase accumulation of NPs in tumors. PEGylation could reduce serum protein adherence and create a stealth surface to prolong the circulating time through avoiding the uptake by the reticuloendothelial systems [12, 17]. The concentration of the encapsulated drugs specifically increased at the tumor sites and significant prolonged enhancement duration time was observed in this study. Therefore, the peak point time of TBR was much later and the imaging latency period was much longer in Omn-NPs group than in Omn group.

Moreover, improved specificity and prolonged enhancement duration time will minimize the injected dose of gadolinium ions, and thus, reducing the risk of nephrogenic systemic fibrosis, a concern in the design of gadolinium-based contrast agents [27, 28].

### IHC Staining for MMP2 and MMP9

**Fig. 5:**
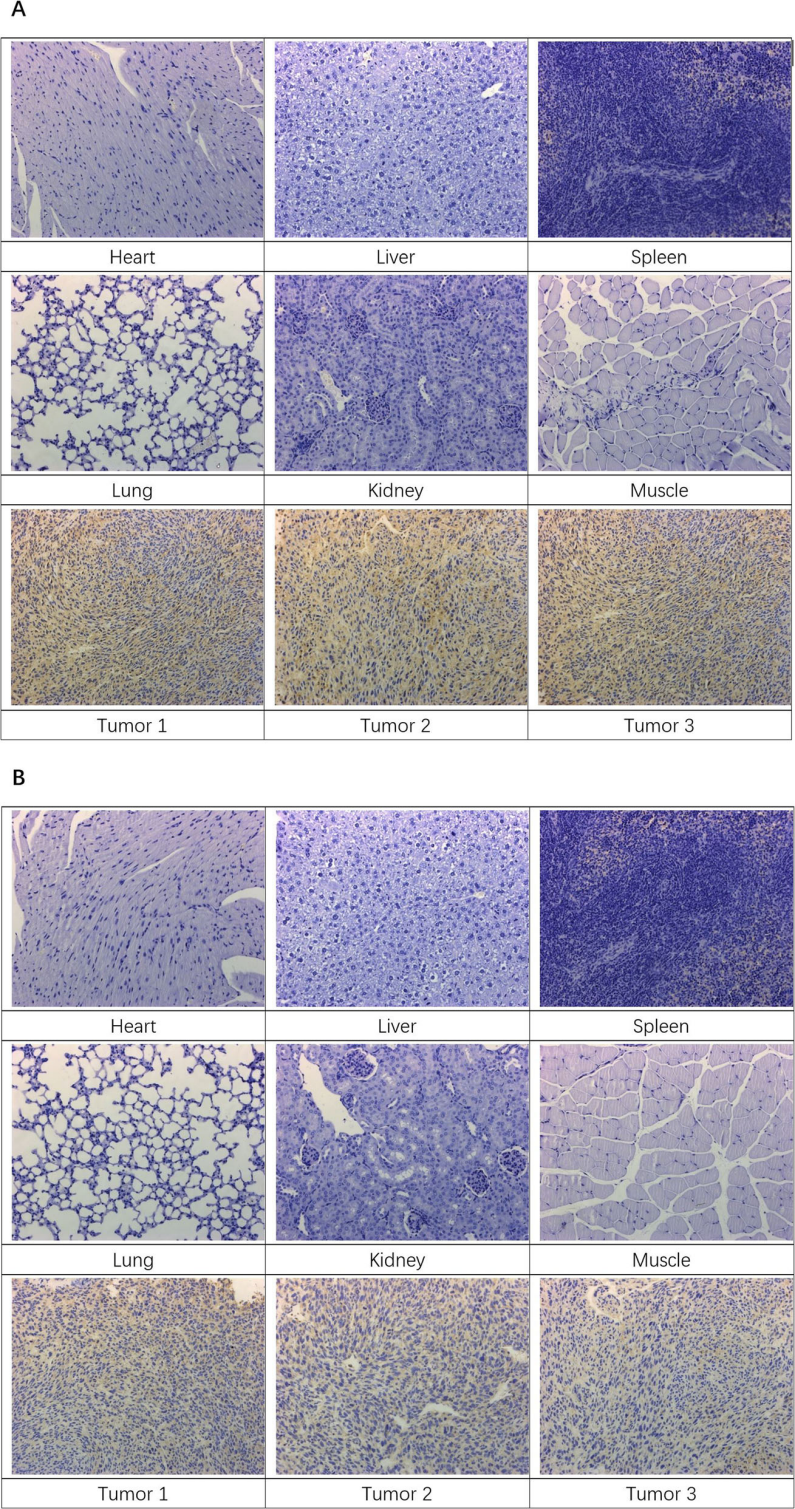
**a**, **b** IHC staining for MMP2 and MMP9 in normal and tumor tissues of xenograft mice model of human oral squamous cell carcinoma lines

The results of IHC staining for MMP2 and MMP9 of normal tissues from organs and tumor tissues are shown in Fig. 5. The results showed the expression levels of MMP2 and MMP9 in tumor tissues were (++), while in most of the normal tissues were (**−**). In the tumor tissues, cell plasma and extracellular matrix were visibly stained brown, indicating higher levels of MMP2/9 expression.

The matrix metalloproteinase (MMP) family, plays a key role in cancer invasion and metastasis, MMP2/9, which are also known as collagenases IV and gelatinases A/B, have been reported to be the most important cancer-related MMPs. Studies have revealed a correlation between gelatinases expression and poor outcomes of many tumors, including OSCC [29, 30]. The high expression of MMP2/9 was also observed in our study. Due to the MMPs are undoubtedly important anticancer targets as their widespread expression and close relation to cancers. Therefore, our Omn-NPs could be used in almost all tumors. Its simplicity and universality has good clinical application potential.

## Conclusions

In this study, we designed and synthesized a novel tumor-targeted MRI contrast agent delivery system to make up for defects of currently used contrast agents in clinic. The higher MRI T1 tumor-to-background ratio and prolonged blood circulation time were found for Omn-NPs than Omn in OSCC mice model. This study demonstrates that Omn-NPs hold promise as tumor-specific MRI contrast agent to improve the specificity and prolong the circulating time in tumor tissues. Considering the excellent biocompatibility and biodegradability properties of PEG and PCL, and Omn is a clinical widely used MRI contrast agent, this tumor-targeted MRI contrast agent delivery system has good clinical application potential. Moreover, further effort will be made on increasing drug loading content and encapsulation efficiency of Omn in our NPs for better sensitivity.

## Data Availability

The data that support the findings of this study are available from the corresponding author on request.

## Abbreviations

NPs: Nanoparticles
Omn: Omniscan
OSCC: Oral squamous cell carcinoma
MMP: Matrix metalloproteinase
Gd: Gadolinium
mPEG: Methoxy-poly(ethylene glycol)
PCL: Poly (ε-caprolactone)
Pep: Peptide
Omn-NPs: Omniscan-loaded mPEG-Pep-PCL NPs
Con-Omn-NPs: Omniscan-loaded mPEG-PCL NPs
MRI: Magnetic resonance imaging
EPR: Enhanced permeability and retention
DCM: Dichloromethane
PVA: Polyvinyl alcohol
DLS: Dynamic light scattering
PDI: Polydispersity index
TEM: Transmission electron microscope
ICP-AES: Inductively Coupled Plasma-Atomic Emission Spectrometry
DMEM: Dulbecco’s modified Eagle’s medium
FBS: Fetal bovine serum
IHC: Immunohistochemical
PBS: Phosphate buffer saline
